# Developing and validating a Women’s Health Index for India

**DOI:** 10.1057/s41271-025-00557-x

**Published:** 2025-02-11

**Authors:** Meena Sehgal, Santosh Jatrana, Louise Johnson

**Affiliations:** 1 School of Humanities and Social Sciences, Faculty of Arts and Education, Deakin University, Geelong, Australia; 2 Alfred Deakin Institute for Citizenship and Globalisation, Deakin University, Victoria, Australia; 3 School of Demography, The Australian National University, Canberra, Australia; 4 Centre for Rural and Remote Health, James Cook University, Mount Isa, QLD Australia

**Keywords:** Women health, Health indicators, Index, Health determinants, India

## Abstract

Developing a women’s health index is particularly important in the Indian context due to the pronounced disparities in women’s health across various social, economic, and geographical segments. This paper focuses on the development and validation of a comprehensive Women’s Health Index (WHI) for India. In contrast to previous efforts that predominantly focused on indicators related to pregnancy, childbirth, and the postpartum period, often limited to reproductive or maternal health services, our index adopts a multi-dimensional approach. The WHI comprises 17 variables, representing life stages of women and encompassing a diverse range of factors. Utilizing Principal Component Analysis, we computed the index and conducted statistical tests for internal and external validation. The spatial pattern of WHI reveals substantial inter-district and inter-state disparities. This study thus provides a valuable tool for characterizing women’s health and identifying health inequalities at a smaller administrative level in India.

**Supplementary Information:**

The online version contains supplementary material available at 10.1057/s41271-025-00557-x.

## Key messages

- The proposed Women’s Health Index (WHI) includes 17 indicators covering different life stages: adolescence, motherhood, and adulthood.
- WHI uses a robust, multi-dimensional statistical methodology with a strong theoretical foundation, incorporating diverse indicators to capture the complexity of women’s health at a small geographical level.
- The spatial pattern of WHI reveals substantial inter-district and inter-state disparities. This ranking can guide policy-making at a small area level.

## Introduction

Health is a complex and multi-dimensional construct which is influenced by the circumstances in which people are born, grow, live, work, and age [1]. However, both, at the conceptual and methodological levels, there is no consensus about how the concept of health is operationalized. As a result, we have many measures of health, and very few comprehensive measures of health status that represent the multi-dimensional nature of health. Recent thinking in the measurement of population health has been to use a single index combining various measures to a complex phenomenon, such as health [2, 3].

The health of girls and women is a matter of concern, because they are disproportionately affected by many negative social determinants of health, such as gender discrimination, poverty, and lack of education [4]. Traditionally, women’s health has been narrowly defined, often focusing solely on maternal reproductive health during pregnancy, childbirth, and the post-natal period [5–7]. However, proponents of women’s health advocate for a broader perspective that encompasses women’s health and well-being throughout their entire lives, considering their multifaceted roles, and not restricting it solely to reproductive health [5–7]. Nevertheless, capturing the multi-dimensional nature of women’s health quantitatively is challenging, because women constitute a heterogeneous group with varying characteristics, depending upon their context and the demographic. Developing a *composite index of women’s health*, based on multiple indicators across time, offers a comprehensive approach at both the individual and country levels. This index can provide a more holistic understanding of women’s health and its progress overtime. Additionally, women’s health is often considered less important, especially in academic literature and often in policy and there is an added urgency to develop this focus and index to keep the balance [8–10].

Developing a women’s health index for India is an important development. India, as a signatory of the United Nations Cairo Program of Action and the subsequent Beijing Conference in 1995, has taken various policy program initiatives, including the National Population Policy in (March 2020) to reaffirm its commitment to advancing women’s health. Findings from our work can contribute significantly toward Sustainable Development Goal 3 (SDG3), which emphasizes ‘ensuring healthy lives and promoting well-being for people of all ages’ [11]. India’s previous attempts at developing a women’s health index have primarily focused on a reproductive health index [12], maternal health service coverage [13], and Coverage Gap Index (CGI) related to maternal and child health services [14, 15]. These studies have predominantly included indicators related to health only during pregnancy, childbirth, and the postpartum period, primarily within the scope of reproductive health or maternal health service coverage. We know that women’s health encompasses a broader spectrum beyond just maternal or reproductive health or maternal health service coverage. Furthermore, these indices have used only state-level data. When assessing women’s health, it is crucial not only to consider different life stages but also to account for local geographical variations that inform public policy [16]. Given the substantial gender disparities in health across India [17–20], any index constructed at an administrative unit above the district-level risks masking areas of disparity and diverse health care needs. Equally important is the need for considering measuring the indicators using the same dataset. This ensures inferences can be drawn confidently, without attributing variations in results to differences in data quality and methodologies.

The aim of this study is to achieve a holistic perspective and better comprehend how various factors contribute to the overall health of women. We developed a Women’s Health Index for India using the National Family Health Survey (NFHS 5), a nationally representative and publicly available data from the most recent round conducted in 2019–2021. We utilized 17 indicators, spanning across adolescence, motherhood, and adulthood to test internal reliability and construct validity of this index. Our selection of indicators was guided by previous research, focusing on variables that could be objectively measured and interpreted with a well-defined hypothetical relationship (either positive or negative) with the Women’s Health Index. We examined the spatial pattern of women’s health index across the 707 districts of India.

## Data and methods

We analyzed household data at the district level from the latest round of the NFHS 5 survey, purposefully designed to provide comprehensive data on all the indicators required for this research and covered all 707 districts in India. It included a sizeable representative sample, consisting of 636,699 households, 724,115 women, and 101,839 men. We selected the district as a unit of analysis due to large intra-state disparities in health outcomes in India [21, 22]. Additional information about the survey can be obtained from the website of National Family Health Survey, India [23].

### Selection of indicators

We adopted a systematic and theoretically grounded approach to identify the domains for the index and the items within those domains [24], guided by the World Health Organization’s Social Determinants of Health (SDOH) approach [1, 25]. The selection of indicators within this framework was guided by several criteria: data availability at the district level from the same data source to ensure consistency and avoid potential problems related to data quality; relevance to women’s health in the context of India; and the selected indicators display variability among districts and inclusion of indicators that could be used for repeated analysis over time, enabling us to monitor and assess changes in women’s health across the country. Furthermore, we included indicators that represented different stages of women’s life, encompassing adolescence, motherhood/reproduction, and adulthood. The selected indicators were further categorized into four domains to capture a wide range of variables, including socio-cultural factors, women’s health, both protective and risk factors impacting women’s health, and health system and services. Table 1 provides detailed description of each indicator included in the final index along with the expected direction of their association with the overall Women’s Health Index.

**Table 1:** Domains, indicators, and their corresponding life stages used for developing Women’s Health Index for districts of India

Domains (Label)	Indicators	Direction of relationship with health	Life stage		
			Adolescence	Motherhood	Adult
Demographic & socio-cultural					
Women’s education	Female population age 6 years and above who ever attended school (%)	+	x	x	x
Sex ratio	Sex ratio of the total population (females per 1000 males)	+	x	x	x
Early pregnancy	Women age 15–19 years who were already mothers or pregnant at the time of the survey (%)	−	x	x	
Women’s health status					
Blood sugar	Blood sugar level—very high (> 160 mg/dl) (%)	−			x
Blood pressure	Mildly elevated blood pressure (Systolic 140–159 mm of Hg and/or Diastolic 90–99 mm of Hg) (%)	−			x
Health determinants—risk factors					
Low Body Mass Index (BMI)	Women whose Body Mass Index (BMI) is below normal	−		x	x
Obesity	Women who are overweight or obese (BMI ≥ 25.0 kg/m^2^)	−		x	x
Anemia	Non-pregnant women age 15–49 years who are anemic	−	x	x	x
Women use tobacco	Women age 15 years and above who use any kind of tobacco (%)	−		x	x
Women health system and policy					
Antenatal care (ANC) visits	Mothers who had at least 4 antenatal care visits (%)	+		x	
Neonatal tetanus	Mothers whose last birth was protected against neonatal tetanus (%)	+		x	
Iron 180 days	Mothers who consumed iron folic acid for 180 days or more when they were pregnant (%)	+		x	
Post-natal care	Mothers who received post-natal care from a doctor/nurse/LHV/ANM/midwife/other health personnel within 2 days of delivery (%)	+		x	
Family planning	Current use of any family planning method (respondent currently married women age 15–49 years) (%)	+		x	
Health worker FP	Health worker ever talked to female non-users about family planning (%)	+		x	
C-section	Births delivered by cesarean section (%)	+			
Skilled health personnel	Births attended by skilled health personnel (%)	+			

### Statistical analysis

We selected indicators that displayed sufficient variability among districts and preferably un-correlated. For instance, an indicator that either all districts possessed (~ 100%) or none possessed (resulting in a zero-standard deviation) shows no differentiation between districts and thus could contribute little to distinguishing the Women’s Health Index. Detailed methodology can be accessed elsewhere [26]. If indicators exhibited a high degree of correlation (greater than 0.90), only one of them was retained for analysis. All indicators were assumed to be associated with women’s health, and the relationship was altered so that the relation was in the same direction. For example, higher prevalence of obesity indicated poorer health, and where this trend did not align, such as in the case of having neonatal tetanus immunization, the reciprocal of the indicator was utilized. Missing values of indicators have been replaced with the national average.

The indicators were log transformed to take care of skewness of the values and then standardized values of these indicators were computed, each variable was centered to zero and scaled to unit variance. Statistical computations using Principal Component Analysis (PCA) [27, 28] were employed to aggregate the selected indicators, thereby generating a comprehensive, multi-dimensional Women’s Health Index (WHI). This allowed for the ranking of districts based on varying levels of women’s health. PCA included generation of a covariance matrix to identify correlations, the eigenvectors and eigenvalues of the covariance matrix to identify the principal components [29]. The final score determined the ranking of districts on WHI, similar to Human Development Index (HDI) grouping countries in to four quartiles [30]. The index was used to group districts into three categories, namely *leading* with WHI value below 234, *intermediate* with WHI value falling between 234 and 470, and *lagging* with WHI value above 470 (with a lower rank corresponding to better women’s health outcomes).

### Internal consistency and external validation

The internal consistency of the Women’s Health Index was tested by Hierarchical Omega to judge the internal consistency of the variables. The closer the coefficient is to one, the better the verification that the variables were homogeneous. A Hierarchical Omega coefficient of ≥ 0.70 is considered highly reliable [31].

In the absence of any existing district-level women’s health index for all the districts of India, testing external validity was a challenge. Therefore, to assess the external validity of Women’s Health Index, Pearson’s correlation coefficient was calculated between the constructed Women’s Health Index and three indicators at the state level: Maternal Mortality Rate (MMR) for (2018–2020), life expectancy (2016–2020), and the Subnational Human Development Index (SHDI) for the same year at the state level was computed. Thus, the state-level rank was computed by calculating the arithmetic average of Women’s Health Index of the district within each state. We used SRS state-level data, to obtain MMR and life expectancy [32]. The SHDI offers a subnational version of the Human Development Index (HDI) [33]. SHDI comprises three underlying sub-indices (for education, health, and standard of living), covering two education indicators (mean years of schooling of adults aged 25+ and expected years of schooling of children aged 6), along with one each for health (life expectancy at birth) and standard of living (Gross National Income per capita, PPP, 2011 US$).

We used statistical software, SAS release: 9.04.01M7P08062020, for all analyses.

## Results

Descriptive statistics of the indicators included in the Women’s Health Index are provided in Supplementary Table S1. On average, 72% of women had attended school; 12% of women used tobacco; and 18%, 23%, and 56% of women were identified to be low BMI, obese, and anemic, respectively. The overall prevalence of high blood sugar and blood pressure was 6% and 13%, respectively. Around 79% of the women received post-natal care; most of the mothers had four antenatal care visits (61%); most births (90%) were attended by skilled health personnel; and a small proportion of deliveries (23%) were through a C-section. Approximately, 66% women had used a method of family planning; early pregnancy was reported by 7%.

Correlation coefficients between 17 indicators included in the Women’s Health Index are shown in Supplementary Table S2. Most indicators exhibited moderate to low correlation, with several indicators showing negative correlations. As expected, obesity was positively correlated with the prevalence of blood sugar (*r*^2^ = 0.66) and blood pressure (*r*^2^ = 0.35). Antenatal care visits were positively correlated with the consumption of iron tablets during pregnancy (*r*^2^ = 0.69), accessing post-natal care (*r*^2^ = 0.69), undergoing C-sections (*r*^2^ = 0.50), and birth conducted by skilled personnel (*r*^2^ = 0.56).

We conducted PCA using all 17 indicators to determine weights for each indicator and to summarize them into a single score, enabling us to compute the rank of the districts. Supplementary Table S3 shows the eigenvalues from the principal component analysis. By examining the scree plot (Supplementary Fig. S1), we determined the point where the slope of the curve clearly leveled off (the ‘elbow’), and using the variance explained (ensuring that at least 5% of variance was accounted for) (Supplementary Table S3), we determined the number of components that should be retained by the analysis. After examining the scree plot, we retained only six components, these six principal components collectively explained nearly 71% of the total variation in the data (Supplementary Table S3). For example, the first principal component explained nearly 29% proportion of the variation, while the second principal component explained nearly 13% of the variance. The third component explained nearly nine percent of the variation, followed by the fourth and fifth components, which accounted for seven and six percent of the variance, respectively. Lastly, the sixth component explained five percent of the variance. The first principal component assigns large (> 0.30 based on the values of eigenvectors in Supplementary Table S3 positive weights to the following indicators: ANC visits, consumption of iron tablets for 180 days during pregnancy, post-natal care, and birth attended by skilled birth personnel. The second principal component allocates large negative weights to low BMI, anaemia and positive weights to health workers ever talked about family planning. The third to sixth principal components highlight positive weights for indicators, including sex ratio, early pregnancy, blood sugar prevalence, anaemia, family planning, tobacco use, and neonatal tetanus injection. We then grouped all districts were into three categories according to the composite score generated for each district with a lower rank corresponding to better women’s health outcomes (see section “Data and methods”).

### Internal consistency and external validation

The computed Omega Hierarchical was 0.86, which suggested modest internal consistency and appropriateness of the PCA methodology; the value of Omega was higher than the suggested value of 0.70. Table 2 presents the Women’s Health Index ranking, MMR, and the coefficient of variation of the WHI for each state. The WHI correlation with MMR was 0.573, indicating a positive relationship between the two and providing evidence of construct validity. To better illustrate this moderately strong positive relationship between WHI and MMR, refer to the scatter plot in Supplementary Fig. S2. We also correlated the Women’s Health Index with the life expectancy and SHDI. The WHI showed a moderate correlation with life expectancy at 0.67 and with SHDI at 0.59, indicating a moderate relation with this widely recognized measure of overall development.

**Table 2:** State-level Women’s Health Index (WHI) (arranged in ascending order of WHI) and state-level Maternal Mortality Ratio (MMR)

State/UT	# of districts	Range	Mean WHI	Rank WHI	Minimum	Maximum	CV	MMR	Rank MMR	Life expectancy	Rank Life expectancy
Goa	2	35	19		1	36	134	NA		NA	
Puducherry	4	65	36		12	77	79	NA		NA	
Lakshadweep	1	0	40		40	40		NA		NA	
Tamil Nadu	32	151	**43**	**1**	2	153	70	**54**	**5**	73.2	**5**
Kerala	14	166	**46**	**2**	4	170	116	**19**	**1**	75	**2**
Chandigarh	1	0	95		95	95		NA		NA	
NCT of Delhi	11	349	109		30	379	92	NA		75.8	**1**
Dadra Nagar Haveli	3	142	144		73	215	49	NA		NA	
Andhra Pradesh	13	271	**170**	**3**	66	337	51	**45**	**4**	70.6	9
Himachal Pradesh	12	280	174		52	332	52			**73.5**	**4**
Haryana	22	618	**176**	**4**	42	660	78	110	12	69.6	14
Sikkim	4	259	210		135	394	58				
Punjab	22	327	**213**	**5**	53	380	41	105	11	NA	
Andaman & Nicobar	3	175	219		147	322	42	NA		NA	
Karnataka	30	567	225	6	3	570	79	69	8	69.8	15
Telangana	31	416	256		89	505	45	**43**	**3**	70	**13**
Gujarat	33	547	264	8	11	558	58	57	7	70.5	11
Odisha	30	504	278	9	38	542	50	119	15	70.3	12
Jammu & Kashmir	20	350	290		108	458	42	NA		**74.3**	**3**
Uttarakhand	13	304	303	10	117	421	28	103	10	70.6	10
Maharashtra	36	552	311	11	78	630	56	**33**	**2**		
West Bengal	20	657	336	12	7	664	48	103	12	72.3	8
Ladakh	2	23	338		326	349	**5**	NA		NA	
Madhya Pradesh	51	590	340	13	33	623	38	173	18	67.4	20
Rajasthan	33	505	398	14	143	648	30	113	13	69.4	18
Chhattisgarh	27	557	432	15	131	688	36	137	16	65.1	22
Uttar Pradesh	75	518	473	16	177	695	26	167	17	66	21
Manipur	9	482	521		207	689	32	NA		NA	
Assam	33	325	542	17	351	676	16	195	19	67.9	19
Tripura	8	395	558		279	674	23				
Jharkhand	24	325	570	18	346	671	15	56	6	69.6	16
Bihar	38	456	574	19	247	703	19	118	14	69.5	17
Arunachal Pradesh	20	246	598		426	672	**11**	NA		NA	
Mizoram	8	135	636		572	707	**8**	NA		NA	
Nagaland	11	98	662		608	706	**5**	NA		NA	
Meghalaya	11	136	677		569	705	**6**	NA		NA	

Five lowest mean WHI, Coefficient of Variation (CV), and MMR values are shown in bold. For life expectancy and rank of life expectancy five highest values are shown in bold

*NA* not available, *UT* union territories

### Women’s Health Index: district- and state-level variations

The state-level WHI rank showed wide differences between states (Supplementary Table S5 and Fig. S3). Of all the states and union territories (UT), Goa from the West and Puducherry, Lakshadweep, Kerala, and Tamil Nadu from the South depict the best women’s health as indicated by their lower rank and lower score. Bihar, Jharkhand, and Uttar Pradesh from the East and Tripura Arunachal Pradesh, Mizoram, Nagaland, and Meghalaya from the Northeast depict the worst women’s health. Tamil Nadu showed low WHI rankings (ranging from 2 to 153), indicating better health outcomes and a low coefficient of variation CV of 70, indicating low inter-district variation. Kerala exhibited low WHI ranking (ranging from 4 to 170), with a moderate coefficient of variation (CV) of 116. Districts in states such as Bihar and Jharkhand have high WHI rankings, signifying poorer women’s health and low CV, suggesting that all the districts within these states cluster within the lower spectrum of health (Supplementary Table S5 and Fig. S4).

**Fig. 1:**
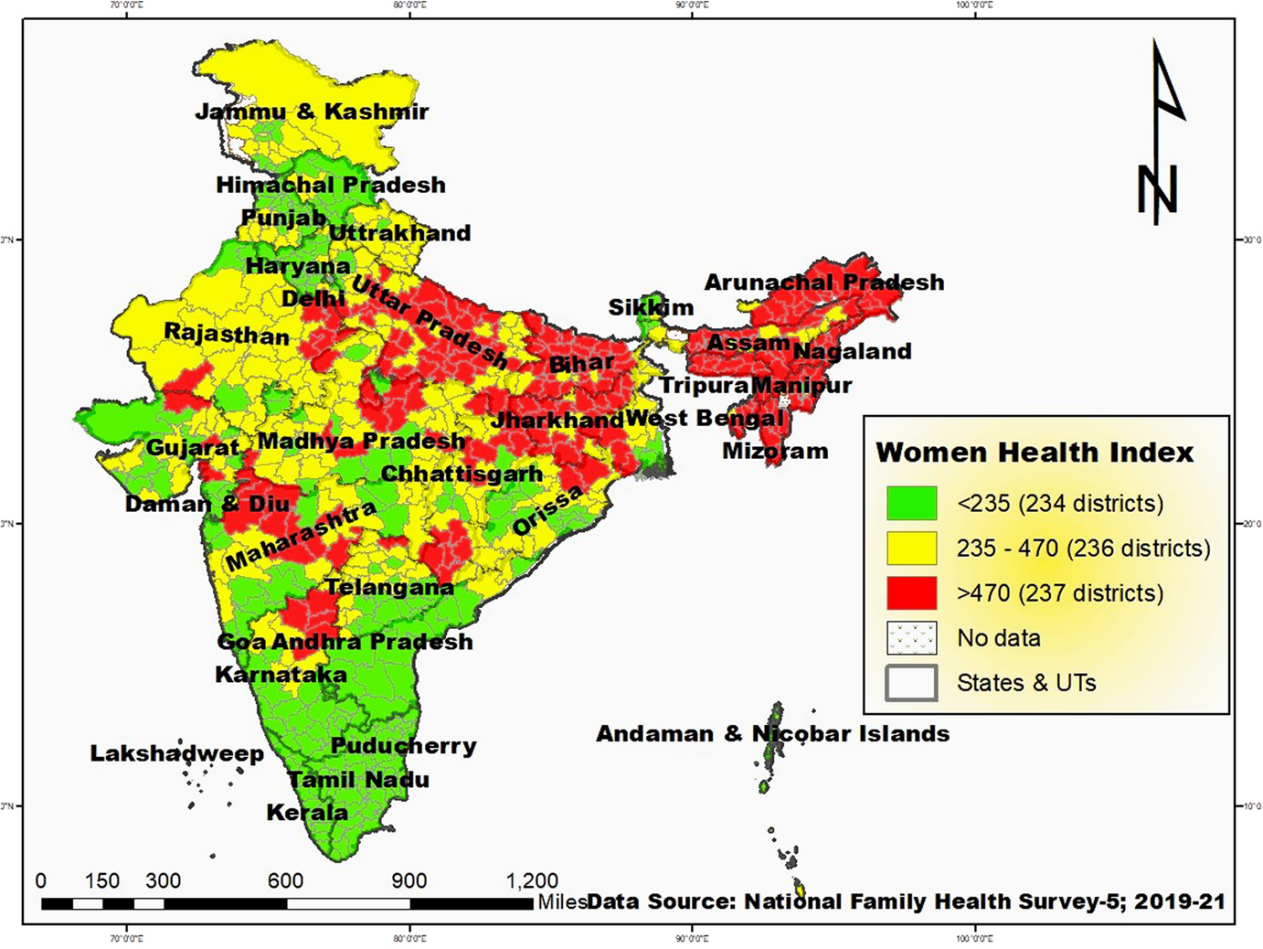
Map for all districts of India classified by ranking of Women’s Health Index

The distribution for all districts of India classified by ranking of Women’s Health Index is shown in Fig. 1. In states like Kerala and Tamil Nadu, all districts are positioned in the lead category, signifying that their WHI values fall below 234. Conversely, in Meghalaya, every district is classified in the lagged category, indicating WHI values exceeding 470. In Ladakh, all districts fall under the intermediate category. Districts in the lead category are primarily distributed across states, with a majority (60%) located in Tamil Nadu (14%), Haryana, and Karnataka (each approximately 7%). Other states such as Gujarat, Maharashtra, Punjab, Kerala, and Telangana also contribute 6% each to this category. A significant portion (60%) of the lagged category comprises districts from Uttar Pradesh (18%), Bihar (14%), Assam (11%), and Jharkhand and Arunachal Pradesh (each with 8% representation).

### Predicting Women’s Health Index

The correlation coefficients presented in Table 3 shows a positive association between key indicators and WHI. Moderate or strong positive correlations with WHI were observed across various indicators in each domain. Specifically, in the health system and policy domain, lower rates of antenatal care, prompt post-natal medical care, and the rate of C-section exhibited strong positive correlations, with respective values of *r*^2^ = 0.83, *r*^2^ = 0.73, and *r*^2^ = 0.62. Conversely, in the socio-cultural domain, women’s schooling demonstrated a negative weak correlation with WHI (*r*^2^ = − 0.34). Health status indicators such as the prevalence of blood sugar and blood pressure showed moderate and weak negative correlations (*r*^2^ = − 0.52 and − 0.39, respectively). Additionally, in the health-determining risk factor domain, obesity displayed a moderate negative correlation with WHI (*r*^2^ = − 0.60).

**Table 3:** Pearson correlation coefficients of district-level Women’s Health Index (WHI) with indicators in each domain

Domains	Variables/indicator	Correlation with WHI	*P* value
Demographic & socio-cultural (or material well-being items)	School	− 0.344	< 0.0001
	Sex ratio	0.051	0.178
	Early pregnancy	0.281	< 0.0001
Women’s health status	Blood sugar	− 0.518	< 0.0001
	Blood pressure	− 0.394	< 0.0001
Health determinants—risk factors	Low Body Mass Index (BMI)	0.171	< 0.0001
	Obesity	− 0.603	< 0.0001
	Anemia	0.024	0.518
	Women use tobacco	0.447	< 0.0001
Women health system and policy	Antenatal care (ANC) visits	0.728	< 0.0001
	Neonatal tetanus	0.366	< 0.0001
	Iron 180 days	0.733	< 0.0001
	Post-natal care	0.837	< 0.0001
	Family planning	0.583	< 0.0001
	Health worker FP	0.215	< 0.0001
	C-section	0.622	< 0.0001
	Skilled health personnel	0.594	< 0.0001

## Discussion

We developed a comprehensive Women’s Health Index at a district level in India, which was validated using Maternal Mortality Ratio as a women’s health indicator, a measure known for its regional gradient [34–36]. To the best of our knowledge, this kind of work has never been undertaken in India or globally, especially at such a refined geographical level. Operating at this scale enhances the uniformity of respondents’ characteristics, enabling us to more precisely identify and measure women’s health. Unlike previous efforts that primarily focused on specific indicators related to reproductive or maternal health services during pregnancy, childbirth, and the postpartum period [12–15], this index adopts a multi-dimensional approach. It encompassed indicators representing various life stages of women and a wide spectrum of factors, including socio-cultural influences, health determinants, and dimensions associated with health systems and policies. These collectively shape women’s well-being. Additionally, these indicators are derived from a single publicly accessible recurring data source, ensuring consistency and comparability across all districts. The recurring nature of this data source opens opportunities for trend analyses regarding women’s health, not only in India but also in other regions where similar data sources are accessible.

There are various approaches for aggregating indicators into an index, such as averaging them as used by the Indian Ministry of Health and Family Welfare [37] method. However, this assumes that all indicators are equally weighted or assigned weights based on expert-defined criteria [38]. A more effective method, which has been employed in several studies, involves the use of factor analysis or Principal Component Analysis to derive a health index [39–41]. We chose to use PCA to aggregate the 17 indicators, a statistical approach that helps assign weights for each indicator. The resulting rankings were useful for comparing districts and classifying them within the index.

The indicators included in the index exhibited a moderate level of internal consistency. The hierarchical omega (0.86) suggested that the PCA methodology was both internally consistent and appropriate for the analysis. Testing external validity was challenging in identifying suitable district-level indicators. As a result, we had to rely on the state-level measure to establish a correlation with WHI. We looked at the correlation between state-level WHI and Maternal Mortality Ratio. It is important to bear in mind that Maternal Mortality Ratio data is only available for larger states. As a measure of single health endpoint, Maternal Mortality Ratio may not fully capture the status of other crucial indicators, particularly in the context of increasing life expectancy and the prevalence of chronic disease. Even state-level estimates can be unreliable due to the small numbers of recorded deaths, given that maternal deaths remain an uncommon cause of mortality [42].

Using WHI, we revealed spatial disparities, highlighting areas where resource allocation and policy development are needed to address both inter-state and intra-state disparities, with the goal of improving women’s health during both motherhood and adulthood. The Women’s Health Index demonstrates a strong positive correlation with key factors, reflecting the effect of the difference in residential areas in growing economies which show a higher prevalence of obesity and blood sugar levels in urban areas [43] but better maternal indicators because of better infrastructure and information [44]. Our findings are consistent with studies from India which have shown significant regional coverage gap in utilization of maternal and child health care services [15, 45, 46]. The Women’s Health Index could serve as a valuable tool for guiding and evaluating health planning, resource allocation, and examining disparities in health to address maternal mortality, given significant interstate variations [34, 36, 47].

### Limitations

We acknowledge that the index has limitations in terms of capturing quality of health care services and overall quality of life. Our analysis could not incorporate these variables due to unavailability of data. The data source, being a nationwide survey rather than a census, means that the rates and prevalence used are estimates. In constructing WHI, we acknowledge that the selection of variables was inherently influenced by the authors’ expert judgment and informed by previous research the availability of reliable data. Our choices were guided by the objective to create a comprehensive and representative index, despite the inherent limitations and data constraints. We have selected indicators that are uniformly collected and scientifically validated in the NFHS 5 survey to ensure consistency and comparability across districts. We recognize that this approach involves a balance between scientific rigor and practical feasibility. We advocate for future iterations of the WHI to encompass indicators related to mental health status and health metrics for the geriatric population, enhancing its comprehensiveness and relevance. Additionally, while developing the index at the district level offers valuable insights into regional disparities, some districts in India are large and populous, potentially containing pockets of significantly different health outcomes. Creating the index at a smaller area level, such as the municipality or block level, could provide a more detailed understanding of local differences and better inform localized interventions. Finally, the study’s findings are based on data from a specific time (NFHS 5), and women’s health outcomes may vary over time due to changes in policies, programs, and socioeconomic factors. Regular updates on the index using recent data are necessary to track progress and identify emerging health issues.

## Conclusion

The developed Women’s health Index for India has several notable strengths that make it a valuable tool for addressing women’s health challenges. This is the first validated tool that we are aware of for ranking women’s health at a district level. Using publicly available data, considering different life stages, and providing district-level comparison, the index allows for a comprehensive assessment of women’s health across the country. Regular monitoring using the index, along with ongoing refinement and validation, can lead to evidence-based policies and targeted interventions to improve women’s health outcomes and promote equity in women’s health in India. Moreover, this index can be modified and replicated in other countries where similar data from Demographic and Health Surveys are available.

## Supplementary Information

Below is the link to the electronic supplementary material.

Supplementary file1 (DOCX 358 KB)

## Data Availability

Not applicable.
