# Supplementary material for: Developing and validating a Women’s Health Index for India
Source: J Public Health Policy. 2025 Feb 11;46(2):342–58. doi: 10.1057/s41271-025-00557-x (PMC12119334; doi:10.1057/s41271-025-00557-x)
Supplement: Supplementary file 1 — Supplementary file1 (DOCX 358 KB) [file 41271_2025_557_MOESM1_ESM.docx]

**Developing and validating a Women’s Health Index for India**

Meena Seghal^1^ *****, Santosh Jatrana^2,3.4^, Louise Johnson^2^

^1^ School of Humanities and Social Sciences, Faculty of Arts and Education, Deakin University, Geelong, Australia

^2^ Alfred Deakin Institute for Citizenship and Globalisation, Deakin University, Victoria, Australia

^3^ School of Demography, The Australian National University, Canberra, Australia

^4^ Centre for Rural and Remote Health, James Cook University, Mount Isa, Queensland, Australia

* Corresponding author: School of Humanities and Social Sciences, Faculty of Arts and Education, Deakin University, Geelong, Australia

[msehga@deakin.edu.au](mailto:msehga@deakin.edu.au)

**Supplementary Material**

**
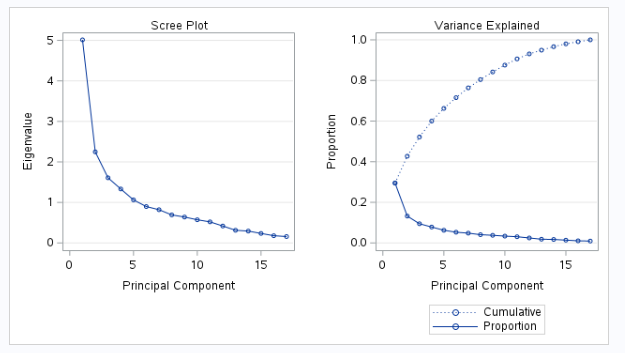
**

**Supplementary** **Fig. S1** Scree plot of eigenvalues plotted against factors for the initial 17 variables used for principal component analysis

**Supplementary Fig. S2** Scatter plot of mean Women’s Health Index (WHI) and Maternal Mortality Ratio (MMR) for the states


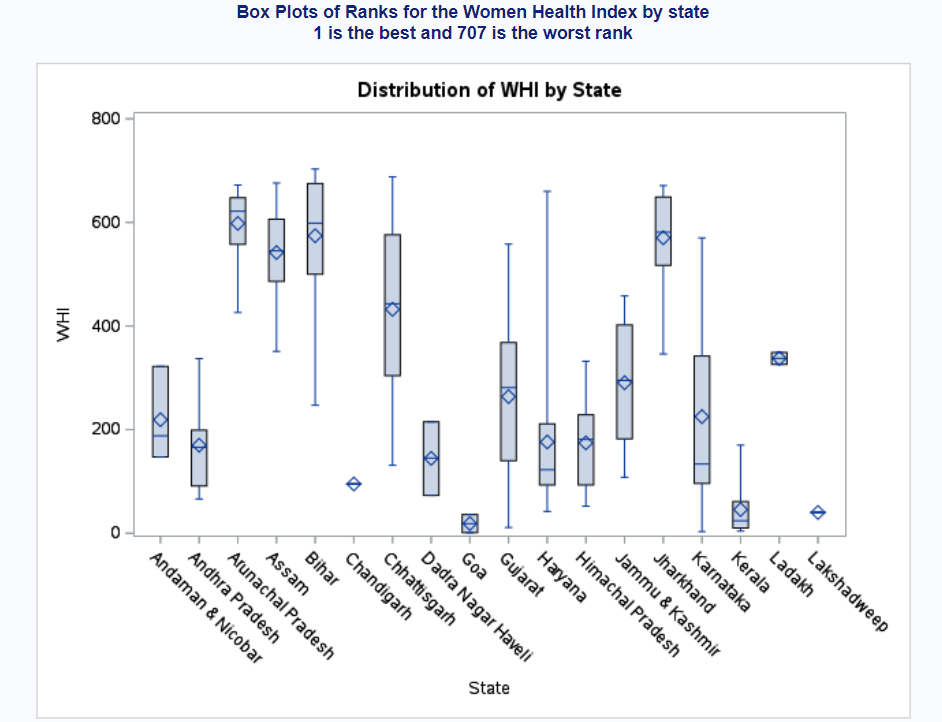


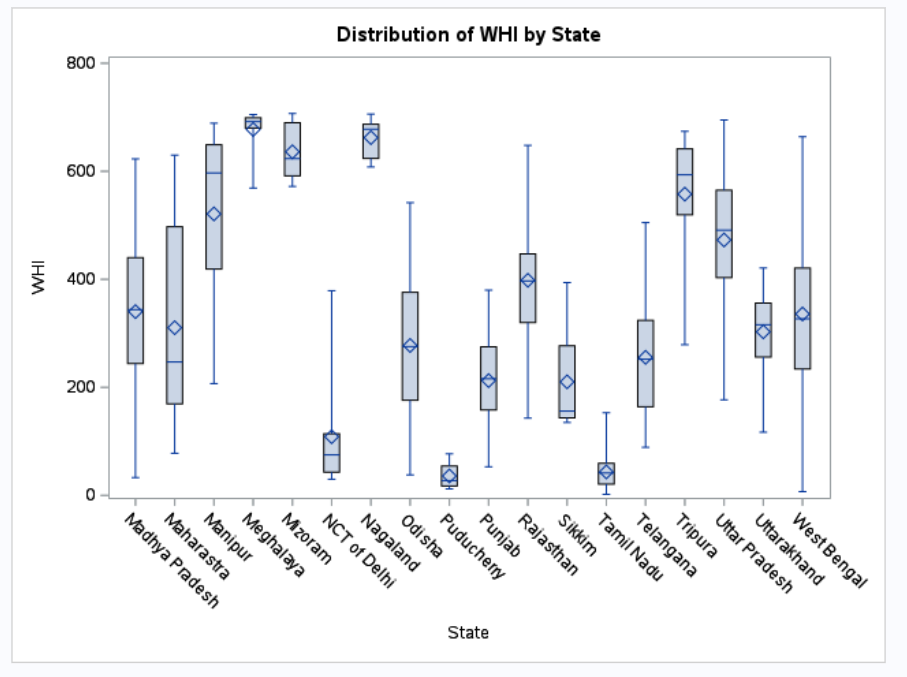


**Fig. S3** Box plots of distribution of Women’s Health Index (WHI) in 36 States and Union Territories (UTs) of India


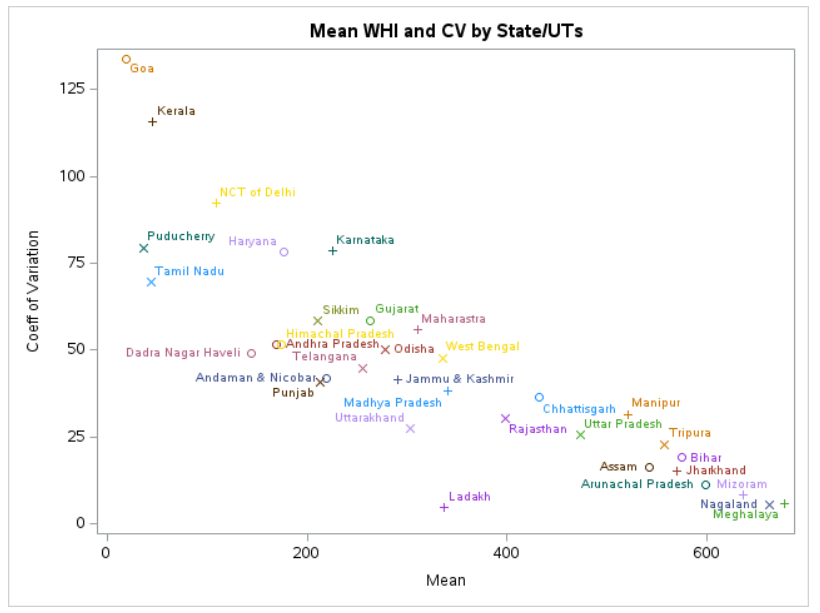


**Fig. S4** Plot of mean Women’s Health Index (WHI) and Coefficient of Variation (CV) for states/ Union Territories. The colours are to aid connect the location of the point to the state/ Union Territories

**Supplementary Table S1** Descriptive statistics for Indicators in the Women’s Health Index (WHI)

| Indicators | Mean | Std Dev | Minimum | Maximum |
| --- | --- | --- | --- | --- |
| Female population attended school (%) Sex ratio of the total population  Early pregnancy (%) Blood sugar (%) Blood pressure (%) Low Body Mass Index (BMI) (%) Obesity (%) Anemia (%) Tobacco use (%) Mothers had 4 antenatal care visits (%) Neonatal tetanus (%) Iron folic acid for 180 days (%) Mothers received postnatal care (%) Any method of family planning (%) Health worker for family planning (%) Births by caesarean section (%) Births by skilled health personnel (%) | 72 1021 7 6 13 18 23 56 12 61 91 27 79 66 24 23 90 | 10 73 5 3 3 7 11 12 12 20 6 18 15 12 10 16 10 | 45 755 1 1 4 1 4 16 1 4 55 1 25 12 2 1 31 | 99 1332 27 18 24 44 53 95 71 99 100 85 100 89 64 82 100 |

**Supplementary Table S2** Pearson correlation coefficients between the indicators in the Women’s Health Index (WHI) for all the 707 districts. Correlation >= 0.35 are in bold

| Pearson Correlation Coefficients, N = 707 | | | | | | | | | | | | | | | | | |
| --- | --- | --- | --- | --- | --- | --- | --- | --- | --- | --- | --- | --- | --- | --- | --- | --- | --- |
| Prob > \|r\| under H0: Rho=0 | | | | | | | | | | | | | | | | | |
|  | School | Sex Ratio | Early pregnancy | Blood sugar | Blood pressure | Low Body Mass Index (BMI) | Obesity | Anemia | Women use tobacco | Antenatal care (ANC) visits | Neonatal tetanus | Iron 180 days | Postnatal Care | Family planning | Health worker FP | C- Section | Skilled health personnel |
|  | 1.00 |  |  |  |  |  |  |  |  |  |  |  |  |  |  |  |  |
| School |  |  |  |  |  |  |  |  |  |  |  |  |  |  |  |  |  |
|  | -0.09 | 1.00 |  |  |  |  |  |  |  |  |  |  |  |  |  |  |  |
| Sex Ratio | 0.01 |  |  |  |  |  |  |  |  |  |  |  |  |  |  |  |  |
|  | -0.16 | 0.15 | 1.00 |  |  |  |  |  |  |  |  |  |  |  |  |  |  |
| Early pregnancy | <.0001 | <.0001 |  |  |  |  |  |  |  |  |  |  |  |  |  |  |  |
|  | **0.39** | 0.22 | 0.07 | 1.00 |  |  |  |  |  |  |  |  |  |  |  |  |  |
| Blood sugar | <.0001 | <.0001 | 0.07 |  |  |  |  |  |  |  |  |  |  |  |  |  |  |
|  | 0.24 | -0.06 | -0.16 | 0.21 | 1.00 |  |  |  |  |  |  |  |  |  |  |  |  |
| Blood pressure | <.0001 | 0.10 | <.0001 | <.0001 |  |  |  |  |  |  |  |  |  |  |  |  |  |
|  | **-0.45** | 0.12 | 0.19 | -0.09 | -0.26 | 1.00 |  |  |  |  |  |  |  |  |  |  |  |
| Low Body Mass Index | <.0001 | 0.00 | <.0001 | 0.01 | <.0001 |  |  |  |  |  |  |  |  |  |  |  |  |
|  | **0.45** | -0.08 | -0.14 | **0.66** | **0.35** | **-0.47** | 1.00 |  |  |  |  |  |  |  |  |  |  |
| Obesity | <.0001 | 0.04 | 0.00 | <.0001 | <.0001 | <.0001 |  |  |  |  |  |  |  |  |  |  |  |
|  | **-0.35** | -0.08 | 0.27 | -0.03 | -0.29 | 0.42 | -0.22 | 1.00 |  |  |  |  |  |  |  |  |  |
| Anemia | <.0001 | 0.04 | <.0001 | 0.37 | <.0001 | <.0001 | <.0001 |  |  |  |  |  |  |  |  |  |  |
|  | 0.04 | 0.05 | 0.17 | -0.18 | 0.04 | 0.07 | -0.31 | -0.04 | 1.00 |  |  |  |  |  |  |  |  |
| Women use tobacco | 0.29 | 0.17 | <.0001 | <.0001 | 0.34 | 0.05 | <.0001 | 0.26 |  |  |  |  |  |  |  |  |  |
|  | -0.29 | -0.09 | 0.04 | **-0.39** | -0.24 | 0.18 | -0.41 | -0.03 | 0.08 | 1.00 |  |  |  |  |  |  |  |
| Antenatal care visits | <.0001 | 0.01 | 0.26 | <.0001 | <.0001 | <.0001 | <.0001 | 0.43 | 0.03 |  |  |  |  |  |  |  |  |
|  | -0.07 | -0.01 | 0.03 | -0.08 | -0.08 | -0.06 | 0.02 | -0.05 | 0.05 | 0.25 | 1.00 |  |  |  |  |  |  |
| Neonatal tetanus | 0.07 | 0.70 | 0.50 | 0.04 | 0.05 | 0.09 | 0.67 | 0.14 | 0.17 | <.0001 |  |  |  |  |  |  |  |
|  | **-0.39** | -0.13 | 0.07 | **-0.54** | -0.34 | 0.09 | -0.45 | 0.08 | 0.21 | **0.69** | 0.19 | 1.00 |  |  |  |  |  |
| Iron 180 days | <.0001 | 0.00 | 0.07 | <.0001 | <.0001 | 0.01 | <.0001 | 0.04 | <.0001 | <.0001 | <.0001 |  |  |  |  |  |  |
|  | -0.20 | 0.02 | 0.21 | **-0.38** | -0.30 | -0.01 | **-0.39** | -0.03 | 0.27 | **0.69** | 0.27 | 0.64 | 1.00 |  |  |  |  |
| Postnatal Care | <.0001 | 0.63 | <.0001 | <.0001 | <.0001 | 0.77 | <.0001 | 0.46 | <.0001 | <.0001 | <.0001 | <.0001 |  |  |  |  |  |
|  | -0.06 | 0.12 | 0.07 | -0.05 | -0.22 | -0.03 | -0.11 | -0.05 | 0.20 | 0.34 | 0.36 | 0.27 | **0.42** | 1.00 |  |  |  |
| Family planning | 0.11 | 0.00 | 0.06 | 0.18 | <.0001 | 0.43 | 0.00 | 0.22 | <.0001 | <.0001 | <.0001 | <.0001 | <.0001 |  |  |  |  |
|  | 0.15 | -0.04 | 0.06 | 0.09 | -0.08 | -0.28 | 0.21 | -0.08 | -0.06 | 0.17 | 0.22 | 0.18 | 0.29 | 0.20 | 1.00 |  |  |
| Health worker FP | <.0001 | 0.26 | 0.11 | 0.02 | 0.04 | <.0001 | <.0001 | 0.03 | 0.11 | <.0001 | <.0001 | <.0001 | <.0001 | <.0001 |  |  |  |
|  | -0.23 | -0.12 | 0.02 | **-0.59** | -0.33 | 0.25 | **-0.64** | -0.05 | 0.24 | **0.50** | 0.08 | **0.48** | **0.48** | 0.12 | -0.13 | 1.00 |  |
| C- Section | <.0001 | 0.00 | 0.59 | <.0001 | <.0001 | <.0001 | <.0001 | 0.16 | <.0001 | <.0001 | 0.04 | <.0001 | <.0001 | 0.00 | 0.00 |  |  |
|  | -0.37 | -0.22 | 0.07 | **-0.47** | -0.23 | 0.21 | **-0.42** | 0.13 | 0.23 | **0.56** | 0.14 | **0.68** | **0.55** | 0.12 | 0.05 | **0.47** | 1.00 |
| Skilled health personnel | <.0001 | <.0001 | 0.06 | <.0001 | <.0001 | <.0001 | <.0001 | 0.00 | <.0001 | <.0001 | 0.00 | <.0001 | <.0001 | 0.00 | 0.16 | <.0001 |  |

**Supplementary Table S3** Eigenvalues of each Principal Components based on PCA. First six eigenvalues that explain over 71 percent of variation are in bold

| Eigenvalues of the Correlation Matrix | | | | |
| --- | --- | --- | --- | --- |
|  | Eigenvalue | Difference | Proportion | Cumulative |
| 1 | **5.013** | 2.765 | **0.2949** | **0.2949** |
| 2 | **2.247** | 0.640 | **0.1322** | **0.4271** |
| 3 | **1.601** | 0.276 | **0.0946** | **0.5217** |
| 4 | **1.332** | 0.270 | **0.0784** | **0.6001** |
| 5 | **1.061** | 0.164 | **0.0625** | **0.6625** |
| 6 | **0.898** | 0.078 | **0.0528** | **0.7154** |
| 7 | 0.820 | 0.128 | 0.0482 | 0.7636 |
| 8 | 0.692 | 0.053 | 0.0407 | 0.8043 |
| 9 | 0.639 | 0.067 | 0.0376 | 0.8419 |
| 10 | 0.572 | 0.051 | 0.0336 | 0.8755 |
| 11 | 0.520 | 0.102 | 0.0306 | 0.9061 |
| 12 | 0.418 | 0.105 | 0.0246 | 0.9307 |
| 13 | 0.313 | 0.020 | 0.0184 | 0.9491 |
| 14 | 0.293 | 0.058 | 0.0172 | 0.9664 |
| 15 | 0.235 | 0.056 | 0.0138 | 0.9802 |
| 16 | 0.179 | 0.021 | 0.0105 | 0.9907 |
| 17 | 0.158 |  | 0.0093 | 1.0000 |

**Supplementary** **Table S4** Principal components for Women’s Health Index showing weights/loadings of each indicator. Large weights are in bold

| Eigenvectors | | | | | | |
| --- | --- | --- | --- | --- | --- | --- |
| Indicators | Principal Component 1 | Principal Component 2 | Principal Component 3 | Principal Component 4 | Principal Component 5 | Principal Component 6 |
| School | -0.245 | 0.293 | -0.091 | 0.171 | 0.200 | -0.147 |
| Sex Ratio | -0.028 | -0.091 | **0.378** | **0.483** | **-0.353** | -0.282 |
| Early pregnancy | 0.084 | -0.127 | **0.460** | 0.088 | **0.484** | -0.146 |
| Blood sugar | -0.309 | 0.048 | **0.362** | 0.007 | 0.036 | -0.010 |
| Blood pressure | -0.214 | 0.104 | **-0.272** | 0.163 | 0.225 | 0.317 |
| Low Body Mass Index (BMI) | 0.160 | **-0.451** | 0.169 | 0.009 | -0.080 | 0.180 |
| Obesity | -0.340 | 0.253 | 0.106 | -0.224 | 0.043 | 0.055 |
| Anemia | 0.082 | **-0.369** | **0.300** | **-0.376** | 0.234 | 0.177 |
| Women use tobacco | 0.137 | 0.043 | -0.018 | 0.530 | **0.535** | 0.229 |
| Antenatal care (ANC) visits | **0.339** | 0.193 | -0.011 | -0.096 | -0.105 | -0.053 |
| Neonatal tetanus | 0.108 | 0.292 | 0.225 | -0.045 | -0.217 | **0.666** |
| Iron 180 days | **0.363** | 0.147 | -0.052 | -0.155 | 0.065 | -0.094 |
| Postnatal Care | **0.335** | 0.275 | 0.117 | 0.058 | 0.103 | -0.172 |
| Family planning | 0.159 | 0.285 | **0.335** | 0.188 | -0.194 | **0.274** |
| Health worker FP | 0.020 | **0.409** | 0.263 | **-0.274** | 0.116 | **-0.290** |
| Births by caesarean section | **-0.343** | 0.035 | 0.191 | -0.221 | 0.155 | 0.110 |
| Skilled health personnel | **0.332** | 0.047 | -0.132 | -0.182 | 0.226 | 0.044 |

**Supplementary Table S5** Women’s Health Index (WHI) ranking at district level grouped into lead, intermediate, and lag category

|  | | Rank | | | Total |
| --- | --- | --- | --- | --- | --- |
|  |  | Lead | Intermediate | Lag |  |
|  |  | < 235 | 235-470 | > 470 |  |
| States/UTs | N | 234 | 236 | 237 | 707 |
| Andaman & Nicobar | N | 2 | 1 | . | 3 |
|  | Column Percent | 0.85 | 0.42 | . | 0.42 |
|  | Row Percent | 66.67 | 33.33 | . | 100.00 |
| Andhra Pradesh | N | 11 | 2 | . | 13 |
|  | Column Percent | 4.70 | 0.85 | . | 1.84 |
|  | Row Percent | 84.62 | 15.38 | . | 100.00 |
| Arunachal Pradesh | N | . | 1 | 19 | 20 |
|  | Column Percent | . | 0.42 | 8.02 | 2.83 |
|  | Row Percent | . | 5.00 | 95.00 | 100.00 |
| Assam | N | . | 7 | 26 | 33 |
|  | Column Percent | . | 2.97 | 10.97 | 4.67 |
|  | Row Percent | . | 21.21 | 78.79 | 100.00 |
| Bihar | N | . | 5 | 33 | 38 |
|  | Column Percent | . | 2.12 | 13.92 | 5.37 |
|  | Row Percent | . | 13.16 | 86.84 | 100.00 |
| Chandigarh | N | 1 | . | . | 1 |
|  | Column Percent | 0.43 | . | . | 0.14 |
|  | Row Percent | 100.00 | . | . | 100.00 |
| Chhattisgarh | N | 3 | 12 | 12 | 27 |
|  | Column Percent | 1.28 | 5.08 | 5.06 | 3.82 |
|  | Row Percent | 11.11 | 44.44 | 44.44 | 100.00 |
| Dadra Nagar Haveli | N | 3 | . | . | 3 |
|  | Column Percent | 1.28 | . | . | 0.42 |
|  | Row Percent | 100.00 | . | . | 100.00 |
| Goa | N | 2 | . | . | 2 |
|  | Column Percent | 0.85 | . | . | 0.28 |
|  | Row Percent | 100.00 | . | . | 100.00 |
| Gujarat | N | 14 | 16 | 3 | 33 |
|  | Column Percent | 5.98 | 6.78 | 1.27 | 4.67 |
|  | Row Percent | 42.42 | 48.48 | 9.09 | 100.00 |
| Haryana | N | 17 | 4 | 1 | 22 |
|  | Column Percent | 7.26 | 1.69 | 0.42 | 3.11 |
|  | Row Percent | 77.27 | 18.18 | 4.55 | 100.00 |
| Himachal Pradesh | N | 9 | 3 | . | 12 |
|  | Column Percent | 3.85 | 1.27 | . | 1.70 |
|  | Row Percent | 75.00 | 25.00 | . | 100.00 |
| Jammu & Kashmir | N | 8 | 12 | . | 20 |
|  | Column Percent | 3.42 | 5.08 | . | 2.83 |
|  | Row Percent | 40.00 | 60.00 | . | 100.00 |
| Jharkhand | N | . | 5 | 19 | 24 |
|  | Column Percent | . | 2.12 | 8.02 | 3.39 |
|  | Row Percent | . | 20.83 | 79.17 | 100.00 |
| Karnataka | N | 18 | 7 | 5 | 30 |
|  | Column Percent | 7.69 | 2.97 | 2.11 | 4.24 |
|  | Row Percent | 60.00 | 23.33 | 16.67 | 100.00 |
| Kerala | N | 14 | . | . | 14 |
|  | Column Percent | 5.98 | . | . | 1.98 |
|  | Row Percent | 100.00 | . | . | 100.00 |
| Ladakh | N | . | 2 | . | 2 |
|  | Column Percent | . | 0.85 | . | 0.28 |
|  | Row Percent | . | 100.00 | . | 100.00 |
| Lakshadweep | N | 1 | . | . | 1 |
|  | Column Percent | 0.43 | . | . | 0.14 |
|  | Row Percent | 100.00 | . | . | 100.00 |
| Madhya Pradesh | N | 12 | 29 | 10 | 51 |
|  | Column Percent | 5.13 | 12.29 | 4.22 | 7.21 |
|  | Row Percent | 23.53 | 56.86 | 19.61 | 100.00 |
| Maharashtra | N | 15 | 11 | 10 | 36 |
|  | Column Percent | 6.41 | 4.66 | 4.22 | 5.09 |
|  | Row Percent | 41.67 | 30.56 | 27.78 | 100.00 |
| Manipur | N | 1 | 2 | 6 | 9 |
|  | Column Percent | 0.43 | 0.85 | 2.53 | 1.27 |
|  | Row Percent | 11.11 | 22.22 | 66.67 | 100.00 |
| Meghalaya | N | . | . | 11 | 11 |
|  | Column Percent | . | . | 4.64 | 1.56 |
|  | Row Percent | . | . | 100.00 | 100.00 |
| Mizoram | N | . | . | 8 | 8 |
|  | Column Percent | . | . | 3.38 | 1.13 |
|  | Row Percent | . | . | 100.00 | 100.00 |
| NCT of Delhi | N | 10 | 1 | . | 11 |
|  | Column Percent | 4.27 | 0.42 | . | 1.56 |
|  | Row Percent | 90.91 | 9.09 | . | 100.00 |
| Nagaland | N | . | . | 11 | 11 |
|  | Column Percent | . | . | 4.64 | 1.56 |
|  | Row Percent | . | . | 100.00 | 100.00 |
| Odisha | N | 12 | 16 | 2 | 30 |
|  | Column Percent | 5.13 | 6.78 | 0.84 | 4.24 |
|  | Row Percent | 40.00 | 53.33 | 6.67 | 100.00 |
| Puducherry | N | 4 | . | . | 4 |
|  | Column Percent | 1.71 | . | . | 0.57 |
|  | Row Percent | 100.00 | . | . | 100.00 |
| Punjab | N | 14 | 8 | . | 22 |
|  | Column Percent | 5.98 | 3.39 | . | 3.11 |
|  | Row Percent | 63.64 | 36.36 | . | 100.00 |
| Rajasthan | N | 3 | 24 | 6 | 33 |
|  | Column Percent | 1.28 | 10.17 | 2.53 | 4.67 |
|  | Row Percent | 9.09 | 72.73 | 18.18 | 100.00 |
| Sikkim | N | 3 | 1 | . | 4 |
|  | Column Percent | 1.28 | 0.42 | . | 0.57 |
|  | Row Percent | 75.00 | 25.00 | . | 100.00 |
| Tamil Nadu | N | 32 | . | . | 32 |
|  | Column Percent | 13.68 | . | . | 4.53 |
|  | Row Percent | 100.00 | . | . | 100.00 |
| Telangana | N | 14 | 16 | 1 | 31 |
|  | Column Percent | 5.98 | 6.78 | 0.42 | 4.38 |
|  | Row Percent | 45.16 | 51.61 | 3.23 | 100.00 |
| Tripura | N | . | 1 | 7 | 8 |
|  | Column Percent | . | 0.42 | 2.95 | 1.13 |
|  | Row Percent | . | 12.50 | 87.50 | 100.00 |
| Uttar Pradesh | N | 4 | 28 | 43 | 75 |
|  | Column Percent | 1.71 | 11.86 | 18.14 | 10.61 |
|  | Row Percent | 5.33 | 37.33 | 57.33 | 100.00 |
| Uttarakhand | N | 2 | 11 | . | 13 |
|  | Column Percent | 0.85 | 4.66 | . | 1.84 |
|  | Row Percent | 15.38 | 84.62 | . | 100.00 |
| West Bengal | N | 5 | 11 | 4 | 20 |
|  | Column Percent | 2.14 | 4.66 | 1.69 | 2.83 |
|  | Row Percent | 25.00 | 55.00 | 20.00 | 100.00 |
